# The tendon unit: biochemical, biomechanical, hormonal influences

**DOI:** 10.1186/s13018-023-03796-4

**Published:** 2023-04-21

**Authors:** Nicola Maffulli, Francesco Cuozzo, Filippo Migliorini, Francesco Oliva

**Affiliations:** 1 Barts and the London School of Medicine and Dentistry, Centre for Sports and Exercise Medicine, Mile End Hospital, Queen Mary University of London, 275 Bancroft Road, London, E1 4DG England; 2 School of Pharmacy and Bioengineering, Keele University Faculty of Medicine, Thornburrow Drive, Stoke On Trent, England; 3 Department of Medicine, Surgery and Dentistry, University of Salerno, Via S. Allende, 84081 Baronissi, SA Italy; 4 Department of Orthopaedic, Trauma, and Reconstructive Surgery, RWTH University Hospital, Pauwelsstraße 30, 52074 Aachen, Germany; 5 Department of Orthopaedic and Trauma Surgery, Eifelklinik St. Brigida, 52152 Simmerath, Germany

**Keywords:** Tendon unit, Tenocyte, Tenoblast, Tendon healing

## Abstract

The current literature has mainly focused on the biology of tendons and on the characterization of the biological properties of tenocytes and tenoblasts. It is still not understood how these cells can work together in homeostatic equilibrium. We put forward the concept of the “tendon unit” as a morpho-functional unit that can be influenced by a variety of external stimuli such as mechanical stimuli, hormonal influence, or pathological states. We describe how this unit can modify itself to respond to such stimuli. We evidence the capability of the tendon unit of healing itself through the production of collagen following different mechanical stimuli and hypothesize that restoration of the homeostatic balance of the tendon unit should be a therapeutic target.

## Introduction

Most of the recent work on the biology of tendons has concentrated on the characterisation of the mechanical, biochemical and biological properties of tenocytes and tenoblasts. It still remains unclear how these cells interact among themselves and are in homeostatic balance with the extracellular matrix (ECM) in which they are embedded in. Focusing on the interactions that occur during mechanical stimuli, under the influence of hormones or growth factors and in pathological states, this brief article aim to explain how this can take place [1]. Tendons connect muscle to bone and allow transmission of forces generated by muscle to bone, resulting in joint movement. Tendon injuries produce considerable morbidity, and the disability that they cause may last for several months de- spite what is considered appropriate management [2].

Regretfully, the pathophysiology of tendon tissue is still poorly understood, and the interactions between the various cell types present in tendons, and between them and the ECM have still not been thoroughly explored. Tendons are multicellular tissue, interposed between bone and muscles, allowing joint movement and stabilisation [3, 4]. Extensive mechanical loads imposed on tendons can lead to acute and chronic injuries [5]. Tendinopathies represent major medical problems associated with overuse, dysmetabolic disorders, inflammation, genetic and familial predisposition, and age-related alteration [1, 6–10]. All these multifactorial agents can contribute to the failed healing response typical of tendinopathic lesions [11, 12].

## Aim

We put forward the concept of a ‘Tendon Unit’ as a metabolic and functional unit of the various cellular components of tendons to at least partially explain how changes in different physiological and pathological conditions may arise from metabolic or (bio)mechanical disarray of such Tendon Unit [13, 14].

## Microanatomical features of the tendon unit

**Fig. 1:**
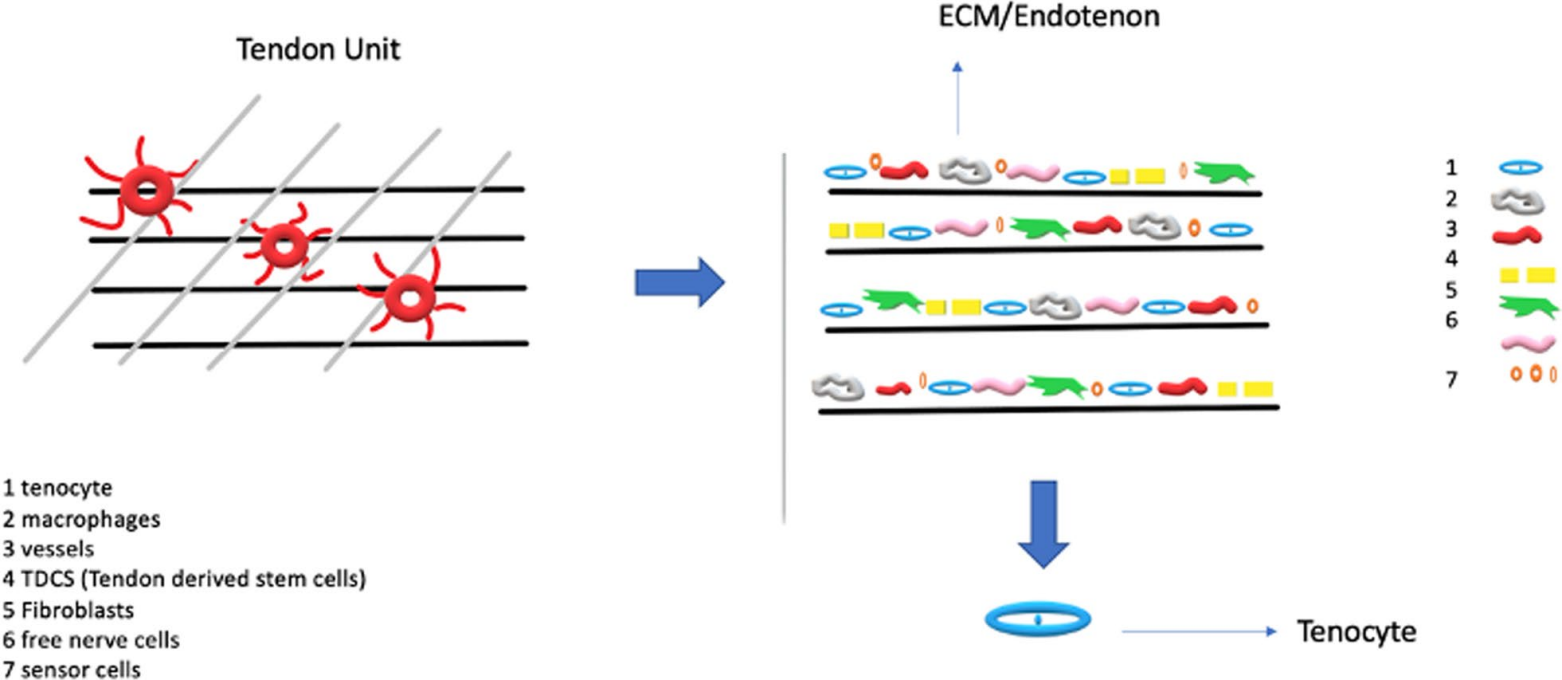
The tendon unit

Tendons are a multi-unit hierarchical structure composed of collagen molecules, fibrils, and fascicles that run parallel to its long axis [15]. Tenoblasts and tenocytes, constitute about 90% of the cellular elements of a tendon [16–18] (Table 1). The other 10% is composed of chondrocytes close to the insertion of the tendon to bone, synovial cells of the tendon surface, vascular cells, such as endothelial cells, smooth muscle cells of the arterioles [3, 19], nerve cells, tendon derived stem cells (TDCS), immune cells [20–22]. Tendons are also surrounded by cell-produced proteins and polysaccharides: collagen (mostly type I collagen) [19, 23, 24], elastin (1–5%) embedded in a proteoglycan (1–5%) and water matrix (70–80%) [24]. The ECM acts as a scaffold, defining the tissue shape and structure, and as a substrate for cell adhesion, growth, and differentiation [25]. Signal transmission of the tendon unit is mediated by the cytoskeleton, integrins, g proteins and stretching-activated ion channel [26]. The cytoskeleton is composed of microfilaments and microtubules and plays a central role in mechanotransduction [27, 28]. Integrins are transmembrane protein heterodimers composed of two subunits. Integrins have three domains: an extracellular matrix domain, a single transmembrane domain, and a cytoplasmic domain [29], playing a role in the signalling interface between the extracellular matrix and the cell [29]. With the integrins, the G proteins are another family of membrane proteins involved in mechanotransduction and are activated by mechanical forces [29]. In addition to the activation of signal proteins, mechanical forces also trigger stretch-activated ion channels [30]. Mechanical stretching induced Ca^++^ signal transmission appears to involve actin filaments, as actin polymerization inhibitors abolished Ca^++^ responses [13] (Fig. 1).

**Table 1:** Main characteristics of tenocytes and tenoblasts

TENOCYTE	TENOBLAST
In the middle of the primary fiber bundle	In the periphery of the primary fiber bundle
Spindle or stellate shape	Round shape cells
Elongated nuclei	Large ovoid nuclei
Condensed cromatin, low concentration of pinocynotic vescicles	High concentration of pinocynotic vescicles
Golgi and endoplasmic reticulum well developed	Golgi and rough endoplasmic reticulum well developed
Many free ribosomes	Few lysosomes
Few mitochondria	Few mitochondria
Predominant in normal tendon	Predominant in young tendon

### Mechanical load and mechanical transduction of the tendon unit

Tendons are exposed to different types of loads during normal function [31], and are subjected mainly to cyclical tensile loads, often working as elastic tissue to decrease the metabolic costs of high-level muscle contraction, exhibiting different behaviours in response to different forces [31, 32]. The tendon unit can adapt to mechanical loading modifying its structure and composition. In particular, the ECM transmits mechanical loads, and stores and dissipates loading-induced elastic energy mediating various cellular functions including DNA and protein syntheses [33]. The adaptative mechanisms whereby tendon units detect mechanical stimuli and modify themselves have only been investigated in vitro [34].

To understand this response, it is important to better define how tendons detect, respond and transduce mechanical stimuli [34]. The changes perceived after mechanical loading are transmitted by gap junctions, which allow between-cell communications: these permit rapid exchange of ions and signalling molecules between cells, inducing stimulatory and inhibitory responses to tensile loads [26], effecting change in the various cellular components of tendons. For example, compressive loading in vitro downregulates SCX, alfa 1 and alfa 2 integrin [35]. These proteins are also significantly downregulated in vivo when tendons are loaded. Short duration compressive loads lead to an increase in the production of type II collagen, aggrecan and lumican [35], while tensile loads lead to an increase of collagen I and III [36]. Hence, modulating both compressive and tensile loads on tendons may prevent a deleterious tendon response modifying collagen production. This should be probably a therapeutic target, but further studies are needed to clarify it [37]. Loading can also influence the production of ECM protein, causing the release of growth factors, such as TGF-b1, bFGF, and PDGF [38]. TGF- b mediates collagen production induced by mechanical loading [39], and also modulates ECM turnover by regulating the expression and activity of MMPs [21, 40–42]. Finally, TGF- b also interacts with growth factors/cytokines to regulate ECM homeostasis in various tissues [43]. Hence, the tendon unit responds to mechanical forces by altering gene expression, protein synthesis, and cell phenotype. These early adaptive responses may influence and lead to long-term tendon structure modifications and thus produce measurable changes in the mechanical properties of tendons [44]. These modified cellular components include the extracellular matrix, cytoskeleton, integrins, G proteins, receptor tyrosine kinases (RTKs), mitogen-activated protein kinases (MAPKs), and stretching-activated ion channels [25].

## Hormones and hormonal disorders

Hormones and hormonal disorders may influence the behavior of the tendon unit [10, 45–47].

A central role is played by oestrogens, thyroid hormones, and relaxin. All the pathological states can influence the tendon healing process and the collagen production.

### Sexual hormones

Oestrogens play an important role in the homeostasis of the tendon unit in pre-menopausal women, who exhibit a lower risk of tendinopathies [48, 49]. Oestrogen levels can influence tendon metabolism, morphology and biomechanical properties [50–52]. Postmenopausal oestrogen deficiency is linked to the down-regulation of the turnover of collagen fibres and a decrease in the elasticity in tendon [53]. Low oestrogen levels are associated with impaired tendon healing with lower cell proliferation, altered metabolism, MMP overexpression, and ECM protein loss [22, 48, 51, 54]. In in vitro models, a decrease in oestrogen levels downregulated collagen turnover and reduced elasticity of tendons [50], and may account for the less favourable outcome experienced by women following surgery for Achilles tendinopathy [55].

### Thyroid hormones

Thyroid hormones (THs) can influence the tendon unit [45, 56]. TH-receptor isoforms are expressed in tendons [46, 47, 57]. Moreover, triiodothyronine (T_3_) and thyroxine (T_4_) contrast apoptosis in healthy tenocytes [46, 58]. THs (especially T_3_) stimulate cellular proliferation and type I collagen formation, the major fibrillar collagen in tendons [46, 58], with an additive effect of ascorbic acid (AA) on T_3_, increasing collagen expression, ECM protein secretion, and the expression of COMP (cartilage oligomeric matrix protein) and tendon cells proliferation [46, 58]. In addition, AA can stimulate the proliferation of tendon cells [58].

### Relaxin

The role of relaxin and how it may influence the tendon unit is not completely clear. Relaxin is a member of a family of peptide hormones structurally similar to insulin, but which diverged from insulin to form a distinct peptide family based on a two-chain structures [59]. Relaxin is antifibrotic and can downregulate fibroblast activity, increase collagenase synthesis, and inhibit collagen I, which is stimulated by transforming growth factor-β (TGFB) [60]. However, the effect of relaxin on tendon and ligament healing remains unclear, although it appears that relaxin inhibits tendon healing [59].

### Glucose metabolism

Diabetes mellitus may be a predisposing factor for tendinopathy [13]. The chronic nature of diabetes demonstrates the long-term effects of elevated glucose levels on tendon cells [13]. Type 2 diabetes mellitus (T2DM) negatively impacts tendon homeostasis in the absence of acute injury [61, 62]. In general, diabetic patients experience an augmented incidence of tendon rupture and tendinopathy [63], with structural abnormalities including calcification [64]. T2DM is a multifactorial pathology, and it is difficult to assess the relative contributions of each factor to diabetic tendinopathy. Elevated serum haemoglobin A1C (HbA1c) levels are strongly associated with the development of the tendinopathy [65, 66]. In terms of the efficacy of tendinopathy treatments, T2DM modifies the response to treatment, with decreased effectiveness [67].

### Growth factors

**Fig. 2:**
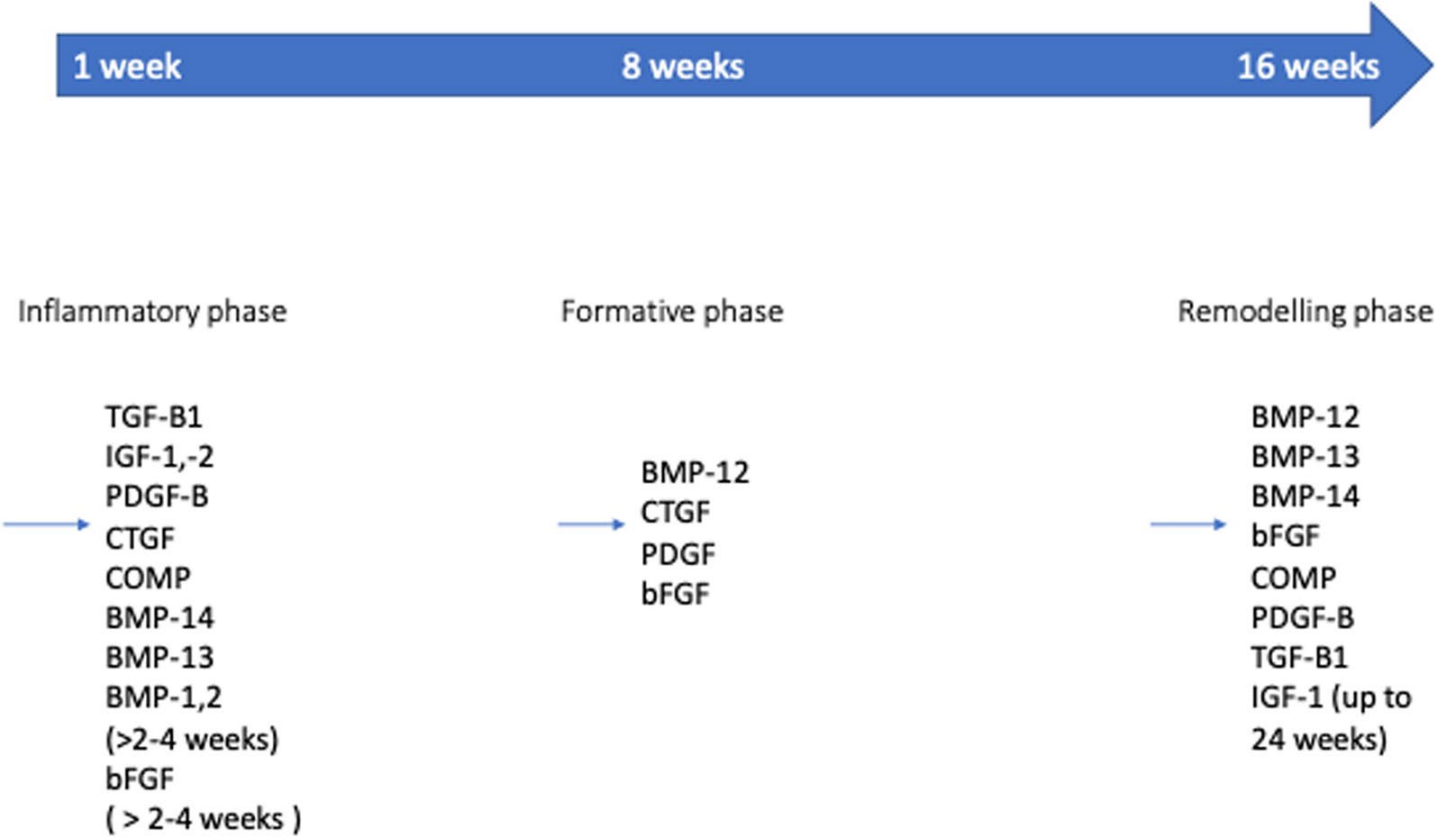
The process of tendon healing goes through three phases. GF is expressed in each phase, promoting the proliferation of cells, ECM and the tendon healing process

Numerous growth factors are involved in the repair processes of the tendon unit [68]. These include BMPs, EGF, FGF1, FGF2, IGF-1, IGF-2, PDGF-AA, PDGF-BB, PDGF-AB, TGF-β, which can influence the tendon unit acting separately or in concert with one another. The expression of the various growth factors is different in each phase of the tendon unit healing process (Fig. 2) [69–72]. The repair process is influenced by inflammation [6, 73].

#### Basic fibroblastic growth factor

bFGF is a single-chain polypeptide of 146 amino acids and is a member of the heparin-binding GF family. bFGF is angiogenic [74], and has mitogenic effects on many mesenchymal cells such as ligament fibroblasts [75]. bFGF is involved in wound healing and exhibits a stimulatory effect on human rotator cuff tendon cells in vitro, though it suppresses collagen synthesis [76].

#### Bone morphogenetic proteins

BMPs (bone morphogenetic proteins) are a group of factors of the TGF-β superfamily that can stimulate formation of bone and stimulate cell mitogenesis and healing in the tendon unit [77], though their mechanism remains unclear [78, 79].

#### Insulin-like growth factor

IGF-1 is found in different cell types, including cartilage, bone, muscle and tendon cells [80]. During the process of tendon healing, IGF-1 seems to stimulate the proliferation and migration of the tenoblasts during the inflammatory phase [81]. In addition to its mitogenic effect, IGF-1 can also stimulate selected components of matrix synthesis and its expression, as seen in vitro in tenocytes [82]. Moreover, in a rat model of Achilles tendon injury, IGF-1 induced tenocyte migration, division, matrix expression and accelerated functional recovery [83, 84].

#### Transforming growth factor-β

Originally known as a tumour transformation factor, it is now clear that TGF-β has a wide range of physiological effects on the tendon unit [85, 86]. The expression of TGF-β seems closely associated with the expression of a differentiated phenotype in some different cell lines, including mesenchymal precursor cells [87]. The formation of tendons and ligaments is directly influenced by the TGF-β superfamily [88]. TGF-β can stimulate tendon cell migration and mitogenesis, but it cannot stimulate robust expression of extracellular matrix [87, 89]. TGF-β, moreover, may control the switching point in the healing process from normal to pathological [90]. All three TGF-β isoforms significantly increase collagen I and III production in cultured tendon fibroblasts [91] with TGF-β_1_ inducing scar tissue formation, whereas TGF-β_3_ reduces it [92].

#### Vascular endothelial growth factors

Vascular endothelial growth factors (VEGF) are two families of proteins resulting from alternate splicing of mRNA from a single, 8 exon, VEGF gene [93]. Probably the most important member is VEGF-A, composed of two subunits. Other members are placenta growth factor, VEGF-B, VEGF-C and VEGF-D [93]. All members of the VEGF family stimulate cellular responses by binding to tyrosine kinase receptors (the VEGFRs) on the cell surface, causing their activation through transphosphorylation [93]. In a canine model of tendon injury, researchers identified a repair site expressing a message for VEGF, suggesting a potential for organizing the angiogenic response during the early postoperative phase of tendon healing [94, 95].

#### Neuropeptides and the tendon unit

The role of substance P (SP) on tendon healing has recently become apparent [96]. Most studies performed on SP and tendons focus on healing after the transection of a tendon [97, 98], but SP may play a role in tendinopathy. In addition to its established role in peripheral pain, SP has pro-inflammatory effects and effects on vasodilation and vascular permeabilization, and also reparative effects including angiogenesis and cell proliferation of the tendon unit [98] (Table 2).

**Table 2:** The main modification of tendon unit according to physiological and pathological states

EXTERNAL STIMULI	CHANGES IN THE TENDON UNIT
Mechanical force	ECM modifier/ TGF B → augmented collagen production
Tyrode hormones	T3: stimulate cellular proliferation, and type I collagen formation, ECM protein secretion, in pathological patterns inhibit tendon healing
Oestrogens	Low: altered ECM metabolism, overexposes MMP and reduce collagen production and tendon healing
Relaxin	Antifibrotic, downregulate fibroblast activity, increase collagenase synthesis, and inhibit collagen I, inhibits tendon healing
Diabetes	Lowers collagen production, inhibits tendon healing
Neuropeptides (P substance)	Accelerates tendon healing, and induces greats angiogenesis

## Future perspectives

Tissue engineering has emerged as a promising approach for tendon healing, with the potential to regenerate functional tendon tissue and improve patient outcomes. Potential future perspectives in tissue engineering for tendon healing include:

1. Advances in biomaterials: Biomaterials play a critical role in tissue engineering by providing structural support and promoting tissue regeneration. New biomaterials are being developed with enhanced mechanical properties, biocompatibility, and bioactivity, which could improve tendon healing outcomes [99].
2. Cellular therapies: Stem cells and other cell-based therapies have shown promise in promoting tendon healing. Researchers are exploring new cell sources and developing new delivery methods to enhance the effectiveness of cellular therapies [100, 101].
3. Bioprinting: 3D bioprinting enables the precise placement of cells and biomaterials to produce complex tissue structures, making it a promising technology for tendon tissue engineering, with new bioprinting techniques and materials to optimize tendon regeneration [102].
4. Gene therapy: Gene therapy has the potential to enhance the healing process by promoting the expression of growth factors and other factors involved in tendon regeneration. Researchers are developing new gene delivery systems to safely and effectively deliver therapeutic genes to damaged tendon tissue [103].

## Conclusions

The tendon unit ensures biosynthesis and the maintenance of the tendon structure. Starting from a mechanical or biochemical stimulus, a series of changes occur in the ECM and the cellular part of the tendon unit. These affect the production of collagen, proteins etc., influencing the tendon healing with a feedback mechanism. Further studies are needed to clarify the molecular mechanism involved in the process of tendon healing and homeostasis. We have concentrated on tenocytes, but tendons are 'organs' with a complex anatomical structure and even more complex physiology. Tissue engineering just using one cell type will have some success, but it is possible that the future will be co-culture of the various cellular components of tendons, coupled with appropriate biochemical and mechanical stimuli, keeping in mind that the tendon unit also contains vascular and neural components.

## Data Availability

Not applicable.

## Abbreviations

ECM: Extracellular matrix
TDCS: Tendon derived stem cells
RTKs: Receptor tyrosine kinases
MAPKs: Mitogen-activated protein kinases
TH: Thyroid hormone
T_3_: Triiodothyronine
T_4_: Thyroxine
AA: Ascorbic acid
COMP: Cartilage oligomeric matrix protein
TGFB: Transforming growth factor-β
T2DM: Type 2 diabetes mellitus
HbA1c: Serum haemoglobin A1C
BMPs: Bone morphogenetic proteins
VEGF: Vascular endothelial growth factors
the VEGFRs: Tyrosine kinase receptors
SP: Substance P
